# AI: the Apollo guidance computer of the Exposome moonshot

**DOI:** 10.3389/frai.2025.1632520

**Published:** 2025-09-10

**Authors:** Fenna C. M. Sillé, Thomas Hartung

**Affiliations:** ^1^Center for Alternatives to Animal Testing (CAAT), Department of Environmental Health and Engineering, Bloomberg School of Public Health and Whiting School of Engineering, Johns Hopkins University, Baltimore, MD, United States; ^2^Doerenkamp-Zbinden-Chair for Evidence-Based Toxicology, Department of Environmental Health and Engineering, Bloomberg School of Public Health and Whiting School of Engineering, Johns Hopkins University, Baltimore, MD, United States; ^3^CAAT-Europe, Department of Biology, University of Konstanz, Konstanz, Germany

**Keywords:** artificial intelligence, Exposome, digital twin, microphysiological systems, multi-omics integration6, human Exposome project, predictive toxicology, systems toxicology

## Abstract

The Exposome—the totality of environmental exposures across a lifetime—remains one of the most significant challenges in understanding and preventing human disease. Translating its vast, heterogeneous data streams into actionable knowledge requires artificial intelligence (AI) integrated with human-relevant experimental systems. We propose a unifying vision in which Microphysiological Systems (MPS) and multi-omics platforms generate high-quality, context-specific data that iteratively calibrate AI models, enabling the creation of digital twins of organs, individuals, and ultimately populations. This “Exposome Moonshot” parallels the Apollo program in ambition, with MPS as the rocket, multi-omics as the lunar module, and AI as the guidance computer. Early applications demonstrate that deep learning can already outperform canonical animal tests for several toxicological endpoints, while reducing cost and time to decision. Realizing the full potential of Exposome intelligence will require expanding the applicability domain of models, implementing robust data security, and prioritizing transparent, interpretable algorithms. By linking predictive AI with experimental feedback, we can move toward a prevention-driven, personalized paradigm for human health and regulatory science.

## 1 Ignition: why exposomics needs an AI engine

Genomics taught us that mapping static code is only half the story; the Exposome—the moving target of everything we breathe, eat, touch, or worry about—drives most of the remaining disease burden (Wild, 2005; Hartung, 2023c). Complex human *in vitro* systems, also known as Microphysiological Systems (MPS), allow modeling these exposures. Yet exposure effects arrive as an unruly torrent of heterogeneous, high-dimensional data. Turning that torrent into knowledge requires artificial intelligence (AI) in the same way the Apollo spaceflight program needed an onboard guidance computer: *“Mass-spectroscopy is our telescope, MPS our lunar module, and AI the guidance computer that stitches the trajectory.”* Extending the analogy, Microphysiological Systems (MPS) can be viewed as the rocket delivering our mission payload. At the same time, multi-omics technologies function as the lunar module enabling precision landing on specific biological questions.

Recently, we have already fused the terms into Exposome Intelligence (EI = Exposome + AI), calling it the “central tool for making sense of ~omics big data” (Sillé et al., 2024; Hartung, 2025). EI is no longer aspirational: industrial-scale machine learning now integrates untargeted mass-spectrometry, wearables, satellite feeds, and electronic health records in near-real time.

## 2 Pattern-finding at planetary scale

Early proof-points show what happens when deep learning meets safety science. Neural networks trained on 600,000 chemicals already outperform the canonical animal tests for skin sensitization, acute toxicity, mutagenicity, and skin and eye hazards, and screen *thousands* of structures in hours (Luechtefeld et al., 2018; Golden et al., 2021; Walter et al., 2024; Duy and Srisongkram, 2025). In the Implementation Moonshot Project for Alternative Chemical Testing (IMPACT) (Sillé et al., 2024), we combine those predictors with evidence-to-decision frameworks^1^ so that regulators can rank hazards and benefits on the same probabilistic scale. Let the algorithms sweat the data so that scientists can sweat the hypotheses.

While AI models show promising performance across diverse toxicological endpoints, their predictive accuracy is ultimately constrained by the applicability domain—defined by the chemical structures, exposure scenarios, and biological contexts represented in their training data. Applicability domain is a concept we introduced earlier (Hartung et al., 2004) for *in vitro* systems, borrowing from the Quantitative Structure-Activity Relationship (QSAR) literature. Still, it is now equally applicable to AI-facilitated New Approach Methods (NAMs), also known as alternatives to animal testing. Current coverage of both chemical space and human-relevant biological responses remains incomplete, particularly for complex mixtures, low-abundance environmental contaminants, and underrepresented population groups. Extrapolation beyond these domains can lead to overconfident or biased predictions (Hartung et al., 2025b). To address these limitations, AI development in exposomics must be coupled to an iterative feedback loop with experimental platforms such as Microphysiological Systems (MPS). In this approach, AI models guide targeted MPS experiments to fill gaps in chemical–biological coverage, while new experimental data are used to recalibrate and extend model applicability. This bidirectional exchange not only improves model robustness and generalizability but also ensures that predictions remain anchored in human-relevant biology, thereby increasing regulatory and clinical confidence in their use.

## 3 Microphysiological systems and their digital twins—the test bed for human digital twins with destination personalization

Fueled by stem cell and sensor technologies (Young et al., 2019), MPS platforms have evolved, which do not only keep single cell types alive and measure cell death, but also replicate aspects of native tissue architecture—such as multi-cellular organization, 3D structure, and barrier function—and physiological functionality, including electrophysiological activity, hormone secretion, and metabolic processing (Roth and Berlin, 2019; Marx et al., 2025; Hartung and Smirnova, 2025). They can act both as human-relevant testbeds and as embodied simulators that refine in-silico models on the fly. So, by creating a digital twin of an MPS, running virtual experiments and based on this refining our twin (Smirnova et al., 2018), we learn how to build the twins for entire humans and then populations. Like this, we are teaching our computers to model organ and whole body responses, so that regulators can finally press ‘quit’ on obsolete animal tests.

In a fully integrated workflow, MPS platforms act as dynamic, human-relevant testbeds that both inform and are informed by AI models. Initial *in silico* predictions, generated from existing chemical–biological data, can be used to prioritize compounds, exposure scenarios, or biological pathways for targeted MPS experimentation. These experiments generate high-content, mechanistically anchored datasets—spanning molecular, cellular, and functional endpoints—which are then fed back into the AI pipeline to refine parameters, extend the applicability domain, and reduce prediction uncertainty. This iterative calibration cycle not only enhances model robustness but also guides the design of subsequent experiments, ensuring that each new data generation step strategically fills knowledge gaps identified by the computational models. Such bidirectional learning aligns with the “systems toxicology” vision, in which experimental and computational tools evolve in concert to progressively approximate human biology while minimizing reliance on animal testing (Smirnova et al., 2018).

The Exposome moonshot ultimately aims at full human digital twins—virtual replicas that integrate genomics, exposomics and clinical trajectories to forecast individual risk and therapy response (De Domenico et al., 2025; Trevena et al., 2024; Gangwal and Lavecchia, 2025). Building such twins depends on advanced modelling plus constant data assimilation, a textbook task for adaptive AI. Key components have been sketched^2^—data fusion, generative modelling, iterative validation. The Exposome Moonshot aims to scale these approaches. If the genome was Apollo 11, the Exposome is Artemis—same audacity, bigger destination.

## 4 AI-driven knowledge creation: faster, cheaper, fairer

Chemical safety testing is traditionally slow, costly and biased toward animal biology. A Human Exposome Project platform uses AI to transform this paradigm (Hartung, 2023a, 2023b; Kleinstreuer and Hartung, 2024). It accelerates data interpretation, slashes costs by automating analysis and reducing animal use, and opens the door to more equitable science by leveraging diverse, real-world datasets—including those from underrepresented populations. An AI-powered Human Exposome Project platform can democratize access to knowledge creation, enabling even low-resource labs and countries to contribute insights. As we expand from chemical safety to exposure sciences and human biomonitoring, AI enables high-throughput, real-time synthesis of data that mirrors how people actually live. Calibrating these outputs against human disease etiology, has the potential to upend medicine as we know it—shifting the gravity from symptom-treatment to prevention and personalization.

In practice, implementing this vision requires integration of: (i) high-resolution environmental exposure data (e.g., air pollutants, dietary profiles, occupational hazards); (ii) biological readouts from minimally invasive sampling (e.g., blood, saliva, hair metabolomics); and (iii) contextual data from wearables and geospatial mapping. MPS platforms can incorporate these datasets by simulating relevant exposure mixtures, using donor-specific induced pluripotent stem cell (iPSC) lines to enable personalized risk modeling. Stem cell-derived MPSs allow individual genetic and epigenetic backgrounds to be represented in exposure-response testing.

## 5 Trust, transparency and the hallucination hang-over

Sceptics fear the “black-box” nature of large models. Encouragingly, hallucination rates in leading large language models have dropped from ~9% to 1–3% in the past year alone, the latest release of Chat-GPT-5 claims 0.7%, and explainability toolkits mature alongside. As Eliezer Yudkowsky warned, “The greatest danger of AI is that people conclude too early that they understand it.” Our task as a community is therefore four-fold:

1. Make models legible—through open weights, provenance metadata and causal audits.
2. Make data FAIR by design—so that learning systems evolve under robust version control (Hartung et al., 2025a).
3. Make the scientific community more AI literate to create a workforce for the Human Exposome and beyond.
4. Using the simplest model when possible ensures highest data security. Where model performance is comparable, simpler and more interpretable algorithms should be favored to facilitate regulatory uptake. Moreover, given the sensitive nature of Exposome and personal health data, the highest standards of data security and privacy must be maintained to ensure public trust and foster data sharing.

## 6 A collective flight-plan

Moonshots are team sports. The Exposome Moonshot Forum (Washington DC, 12–15 May 2025) convened AI researchers, exposure and environmental health scientists, regulators, policy-makers, ethicists and the public to co-author a “Declaration toward a Human Exposome Project.” Working groups shall tackled now critical priorities—from exposomics reporting standards and model cards for safety AI to avenues for public–private co-funding. Regulators aren’t just kicking the tires of new approach methods anymore—they are asking for the keys.

## 7 Conclusions: lighting the next-gen engines

AI has progressed to the point where dismissing it as futuristic is no longer tenable. While some AI domains—such as large language models—are currently doubling in specific performance benchmarks every few months, domains such as exposure science, genomics, and chemistry advance at different rates due to data generation constraints. By the time this reaches print, today’s algorithms will already look vintage. What matters is how quickly we embed them in transparent, ethical and human-centric frameworks.

*“You will never see an AI as bad as today’s—tomorrow’s will be twice as smart and half as hallucinogenic,”* we told journalists recently at an Science Media Centre (SMC) London media briefing. The same exponential curve that propels model capability can, if we steer wisely, propel the Exposome moonshot from concept to clinical and regulatory reality. So let us strap in, check the boosters, and light the engines—Exposome intelligence is ready for lift-off.

## Data Availability

The original contributions presented in the study are included in the article/supplementary material, further inquiries can be directed to the corresponding author/s.

## Footnotes

^1^Available online at: https://www.ebtox.org.

^2^Available online at: https://digital-strategy.ec.europa.eu/en/policies/virtual-human-twins.
